# Clinical efficacy of Stereotactic Body Radiation Therapy (SBRT) for adrenal gland metastases: A multi-center retrospective study from China

**DOI:** 10.1038/s41598-020-64770-2

**Published:** 2020-05-12

**Authors:** Xianzhi Zhao, Xiaofei Zhu, Hongqing Zhuang, Xueling Guo, Yongchun Song, Xiaoping Ju, Ping Wang, Zhiyong Yuan, Huojun Zhang

**Affiliations:** 1 Department of Radiation Oncology, Shanghai Changhai Hospital, Naval Medical University, Shanghai, 200433 China; 2 Department of Radiation Oncology, Peking University Third Hospital, Beijing, 100191 China; 3 Department of Radiation Oncology and CyberKnife Center, Key Laboratory of Cancer Prevention and Therapy, Tianjin’s Clinical Research Center for Cancer, Tianjin Medical University Cancer Institute and Hospital, National Clinical Research Center for Cancer, Tianjin, 300060 China

**Keywords:** Radiotherapy, Physics, Quality of life, Cancer therapy

## Abstract

To evaluate the efficacy and safety of CyberKnife Stereotactic Body Radiation Therapy (SBRT) in the treatment of adrenal gland metastases (AGM), we designed a large-scale multicenter retrospective study to report the safety and efficacy of SBRT for inoperable AGM. In this study, 75 (61 males, 14 females) patients with 84 AGM and Karnofsky performance score ≥70 were treated by SBRT from October 2006 to January 2017. Of these, the purpose of treatment were controlling all known metastatic sites for 21 patients while 54 for palliation of bulky adrenal metastases. The efficacy and safety of SBRT were evaluated during follow-up. Potential factors predictive of local control (LC) and overall survival (OS) were identified by univariate and multivariate analysis. Median follow-up time was 12.7 months (range 1.8–96.4). The complete response (CR), partial response (PR), stable disease (SD) and progressive disease (PD) rates were 23.8%, 23.8%, 31.0% and 21.4%, respectively. The 0.5, 1, and 2-year LC rates were 93.6%, 83.8%, and 62.1%, respectively; OS rates on the same follow-up intervals were 93.7%, 62.5%, and 49.6%, respectively, and the corresponding PFS rates were 48.5%, 33.9%, and 16.0%, respectively. The treatment was well tolerated with 2 patients reporting grade-3 diarrhea and fatigue, respectively. Multivariate analysis showed that simultaneous treatment of SBRT for other metastatic lesions, the number of AGM, initiation of systemic therapy, and the maximum diameter of AGM were predictive of LC rates. Moreover, patients with AGM < 5 cm had a superior OS compared with those with AGM ≥ 5 cm (28.0 months vs. 17.6 months, P = 0.032). SBRT is an effective therapeutic option for treatment of AGM with high LC rates with minimal toxicity.

## Introduction

The adrenal gland is one of the commonest metastatic sites in primary tumors of lungs, liver, renal, colorectal, and lymphoma^1^. Patients with adrenal gland metastases (AGM) have back or abdominal pain occasionally along with the growth of tumor^2^. Rarely, patients with bilateral adrenal metastases may occur adrenal insufficiency which compromises quality of life (QOL) and results in worse outcomes^2^. Although salvage chemotherapy and surgery are commonly used to treat AGM, limitations are inevitable. It is mandatory that pathological examinations of the primary tumor should be performed to guide decision making of chemotherapy regimens. Nevertheless, second-line chemotherapy may be less effective or may contribute to severe toxicities when the disease progresses after first-line chemotherapy has failed. Moreover, surgical resection may bring about adrenal insufficiency or other morbidities albeit it is regarded with curative intent^3^. Hence, radiotherapy may be an alternative due to its potential efficacy and less risk of adverse effects compared to chemotherapy and surgery.

Recent studies have demonstrated survival benefits from stereotactic body radiation therapy (SBRT) for metastatic lesions. Because of its precise delivery of ablative doses to tumors with sparing surrounding normal tissues and organs, good LC could be achieved with mild adverse effects, which may be more suitable for patients with metastases and not eligible for adrenalectomy^4–6^. Till now, it has been used as a modality for treating lung cancer, liver carcinoma, pancreas cancer, and prostate cancer, etc^7–10^. Therefore, SBRT is becoming more and more important in the management of oligometastatic diseases, such as prostate cancer with oligometastatic lymph node invasion, oligometastatic ovarian cancer, or oligometastatic lung cancer^11–13^. However, its clinical utility in the treatment of AGM is rarely reported. So, we conducted this multi-center study to evaluate the efficacy and safety of SBRT in the management of AGM.

## Material and Methods

### Patient characteristics

Seventy-five patients with 84 AGMs were treated with SBRT. SBRT was given for treatment of AGMs in 75 patients (84 target lesions in total) between October 2006 and January 2017 at Tianjin Medical University Cancer Institute Hospital and Changhai Hospital affiliated to Naval Medical University. All patients received detailed reviews about the potential toxicity and benefits of SBRT by the physicians. Patients with AGM were confirmed by biopsy or evaluated by at least one imaging examination (CT, MRI or PET). Patients enrolled were also required to have a Karnofsky performance score over 70, an expectation of life of more than 3 months, and not eligible for surgical resection due to impaired cardiopulmonary functions or other morbidities. Hence, participants would receive personal interviews with physicians for a detailed explanation of related treatments. In addition, written informed consents had been obtained from all patients prior to the treatment, stating their willingness to be treated according to the regimens. The retrospective study was approved by the institutional review board of Tianjin Medical University Cancer Institute and Hospital, and Shanghai Changhai Hospital, and the methods were all in accordance with the Declaration of Helsinki.

### Delivery of SBRT

For those included patients, 21 received relative ablative doses for all metastases while the rest 54 only underwent palliative doses for AGMs due to large tumor sizes or poor medical conditions. Each patient was immobilized in a vacuum bag in supine position. An enhanced CT was performed for target delineations, and the thickness of a slice was 1.5 mm. The range of the scan was from 10 cm below to 10 cm above the tumor. Gross tumor volume (GTV) was the lesion identified by imaging examinations. A previous study11 has clarified that a 3-5 mm and 5–7 mm margin expansion from GTV in lateral and anteroposterior, cephalo-caudal direction was required in the case of X-sight spine tracking and Synchrony™ Respiratory tracking system (Accuray Incorporated, Sunnyvale, CA), respectively. Respiratory tracking system was used in 23 patients while 52 patients received X-sight spine tracking. Physical paramenters of SBRT were shown in Table 1. Dose constraints of organs at risk were demonstrated in Supplementary Table 1 ^12^.

**Table 1:** Treatment parameters used for SBRT.

	All lesions	Lesions withlocal control
GTV (ml)	24.8 (0.25–309.31)	25 (0.25–309.31)
Maximum dose (Gy)	57.55 (43.2–73.2)	57.7 (43.2–73.2)
Total prescribed dose (Gy)	42 (32–52)	42 (32–52)
Number of fractions	5 (3–10)	5 (3–10)
Dose per fraction (Gy)	8 (3.65–15)	8 (3.65–15)
BED10 (Gy)	79.6 (44.8–112.5)	73.9 (44.8–112.5)
Prescription isodose line (%)	72 (61–85)	71 (61–85)

**Figure 1:**
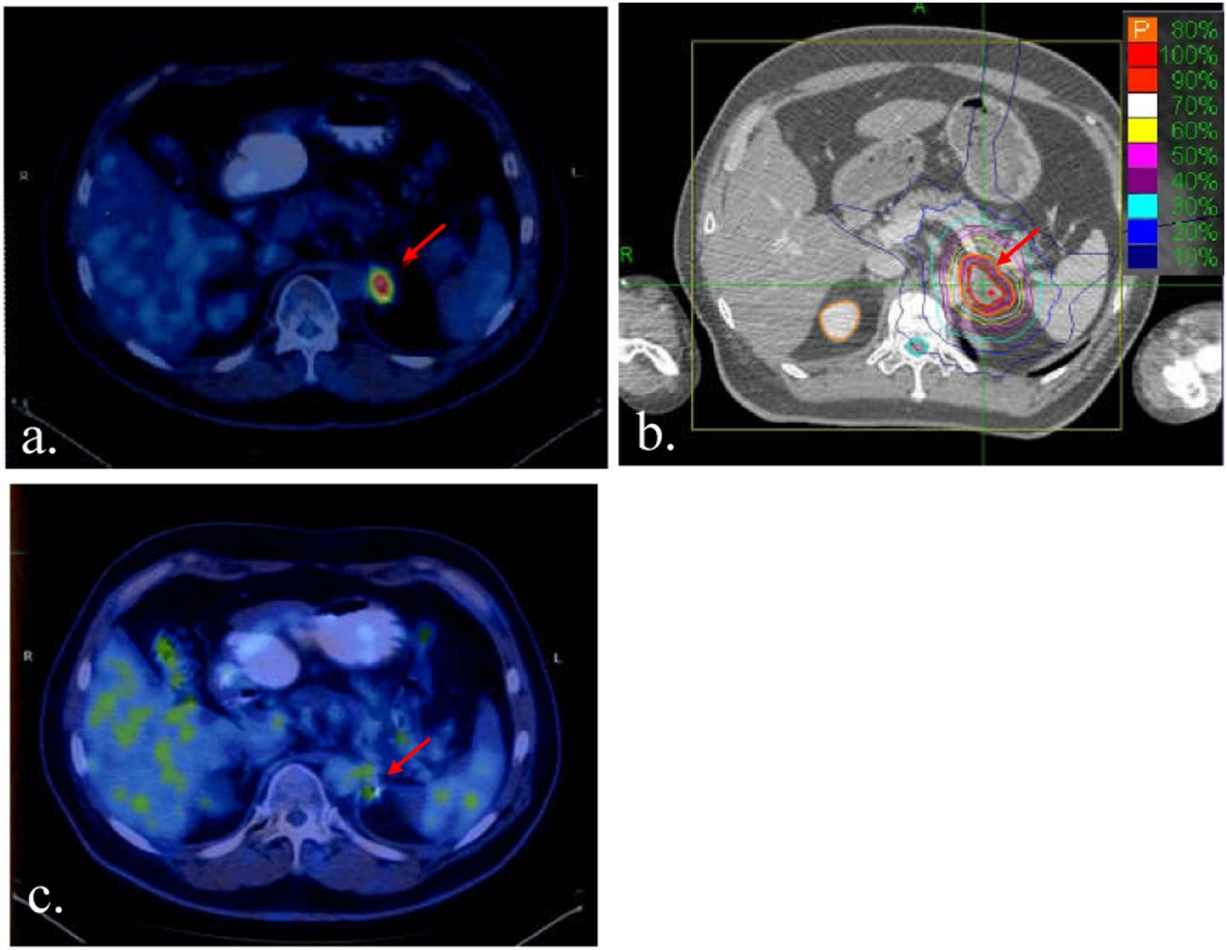
Example of dose distribution and treatment outcome. (**a**) PET-CT scan before SBRT. The arrow shows that the SUVmax of the adrenal metastasis is 8.4 (**b**) Treatment plan with 45 Gy in 3 fractions. The arrow shows that the isodose line is 80% (**c**) PET-CT scan three months after SBRT. The arrow shows no radioactive concentration in the lesion after SBRT.

The median dose was 42 Gy (range: 32–52 Gy) in 5 fractions (range: 3–10 fractions), with the corresponding median biological effective dose (BED_10_, α/β = 10) of 79.6 Gy (range: 44.8–112.5 Gy). The parameters were summarized in Table 1. A case receiving 45 Gy/3 f was shown in Fig. 1.

### Follow-up and data collection

Patients were required to receive the follow-up every 3 months based on imaging examinations including enhanced CT or MRI, or PET-CT, if deemed necessary. Radiation-induced acute and late toxicity were determined by the Common Terminology Criteria for Adverse Events (CTCAE) version 4.0^13^.The definition of tumor response was the response to the treatment, including complete response, partial response, stable disease and progression disease determined by RECIST criteria, version 1.1^14^. LC was no tumor progression after treatment. OS was the time period from the initiation of SBRT to the death by any cause or the last follow-up. PFS was the time period from the initiation of SBRT to identifications of progressions of any tumor sites or death or the last follow-up.

### Statistical analysis

Factors with P values <0.05 in univariate analysis were entered in multivariate analysis to identify predictors correlating with OS and PFS. Factors correlating with OS ad LC were identified by univariate and multivariate analysis. OS and LC were calculated by Kaplan-Meier method and compared between different groups by log-rank test. Statistical analyses were performed by SPSS version 18.0, and two-sided P values <0.05 were regarded as statistical significance.

## Results

### Patient characteristics

Patient characteristics were presented in Table 2. Seventy-five patients receiving SBRT were included. Of all patients, 31 had the tumor in the left adrenal gland while 35 and 9 had right AGM and bilateral AGM, respectively. The origin of primary tumor included the lungs (n = 30, 40.0%), liver (n = 20, 26.7%), kidney (n = 7, 9.3%), colorectum (n = 5, 6.7%), non-Hodgkin lymphoma (4/5.3%), stomach (n = 2, 2.7%), esophagus (n = 2, 2.7%), pancreas (n = 2, 2.7%), adrenal cortical adenocarcinoma (n = 1, 1.3%), nasopharynx (n = 1, 1.3%), and breast (n = 1, 1.3%). Among all the patients, 38 (50.7%) had other metastases in addition to adrenal lesions. 46 (61.3%) had received chemotherapy before or/and after SBRT. SBRT was performed for more than one lesion in 49 patients (65.3%).

**Table 2:** Patient demographics and clinical presentation.

Characteristics	Values		
Age (years)	58 (range 27–85)		
Gender (male/female)	61/14 (81.3%/18.7%)		
Karnofsky performance score ≥70	75 (100%)		
**Primary tumor**		**Symptoms**	
▪ Lung cancer	30 (40.0%)	▪ Presented	36 (48.0%)
▪ Liver cancer	20 (26.7%)	▪ None	39 (52.0%)
▪ Renal cancer	7 (9.3%)	**Lesions per patient**	
▪ Colorectal cancer	5 (6.7%)	▪ 1	66 (88.0%)
▪ Non-Hodgkin lymphoma	4 (5.3%)	▪ >1	9 (12.0%)
▪ Gastric cancer	2 (2.7%)	**Chemotherapy**	
▪ Esophageal cancer	2 (2.7%)		
▪ Pancreatic cancer	2 (2.7%)	▪ Yes	46 (61.3%)
▪ Adrenal cortical adenocarcinoma	1 (1.3%)	▪ No	29 (38.7%)
▪ Nasopharyngeal carcinoma	1 (1.3%)	**Metastases of other sites**	
▪ Mammary cancer	1 (1.3%)	▪ Yes	38 (50.7%)
**Location of adrenal metastases**		▪ No	37 (49.3%)
▪ Left	31 (41.3%)	**SBRT of other sites**	
▪ Right	35 (46.7%)	▪ Yes	49 (65.3%)
▪ Left and right	9 (12.0%)	▪ No	26 (34.7%)

### Outcomes

**Figure 2:**
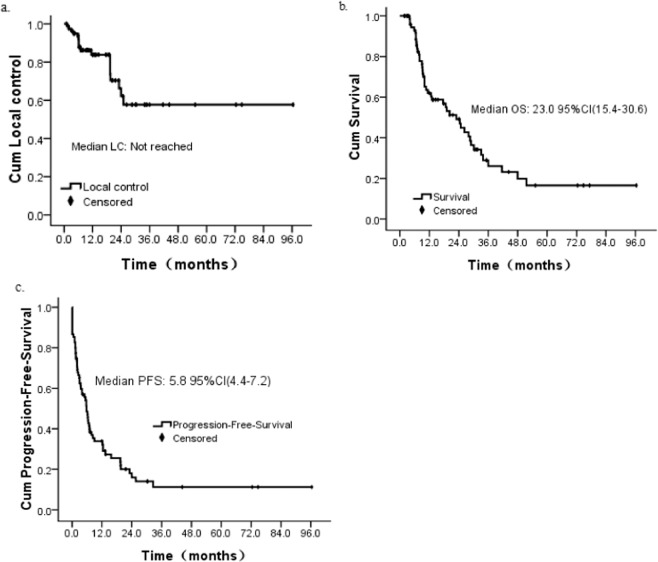
Survival probability analysis of patients. (**a**) Overall local control. (**b**) Overall survival in general. (**c**) Overall progression-free-survival. Cum, cumulative.

At the last follow-up, 45 patients (60.0%) died while the rest 30 patients were alive. The median follow-up was 12.7 months (range 1.8–96.4 months), median OS was 23.0 months (95%CI: 15.4–30.6months), and median PFS was 5.8 months (95%CI: 4.4–7.2months). The 6-month, 1-year, and 2-year LC rates were 93.6%, 83.8%, and 62.1%, respectively (Fig. 2a). Similarly, the 6-month, 1-year, and 2-year OS rates were 93.7%, 62.5%, and 49.6%, respectively (Fig. 2b), and the corresponding PFS rates were 48.5%, 33.9%, and 16.0%, respectively (Fig. 2c). Using the RECIST criteria, the CR, PR, SD and PD rates were 23.8%, 23.8%, 31.0% and 21.4%, respectively.

**Figure 3:**
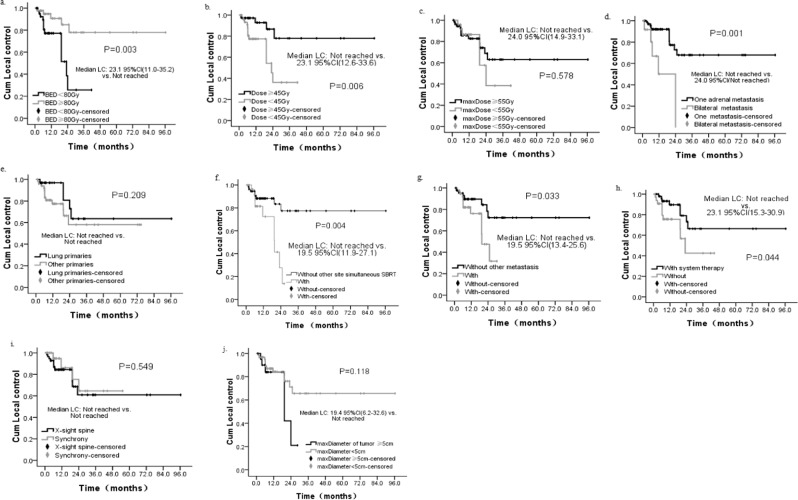
Local control probability of patients. (**a**) Local control depending on biologic equivalent dose (BED10). (**b**) Local control depending on prescribed dose. (**c**) Local control depending on max dose to tumor. (**d**) Local control depending on involved adrenal gland (AG) per patient. (**e**) Local control depending on histological primaries (**f**) Local control depending on with other site SBRT simultaneously. (**g**) Local control depending on concurrence of other site metastasis. (**h**) Local control depending on prior or/and after chemotherapy. (**i**) Local control depending on tracking method. (**j**) Local control depending on the maximum diameter of AGM. Cum, cumulative.

Based on the univariate analysis, patients with BED_10_ ≥ 80 Gy, prescription dose ≥45 Gy, one AGM, without simultaneous SBRT of other sites, without other metastases and with systemic therapy had better LC compared to those with BED_10_ < 80 Gy (Not reached vs. 23.1 months, P = 0.003) (Fig. 3a), prescription dose<45 Gy (Not reached vs. 23.1 months, P = 0.006) (Fig. 3b), bilateral AGM (Not reached vs. 24.0 months, P = 0.001) (Fig. 3d), with simultaneous SBRT of other sites (Not reached vs. 19.5 months, P = 0.004) (Fig. 3f), with other metastases (Not reached vs. 19.5 months, P = 0.033) (Fig. 3g), and without systemic therapy (Not reached vs. 23.1 months, P = 0.044) (Fig. 3h). In the multivariate analysis of LC rates, with/without simultaneous SBRT of other sites (HR = 4.324, 95%CI (1.613–11.590), P = 0.004), and the number of AGM (HR = 4.907, 95%CI (1.787–13.474), P = 0.002) were independent prognostic factors. Furthermore, patients with different max-Dose (P = 0.578) (Fig. 3c) or different origins of the primary tumors (P = 0.209) (Fig. 3e) had similar LC. Also, different tumor tracking methods of SBRT did not influence the LC (P = 0.549) (Fig. 3i).

**Figure 4:**
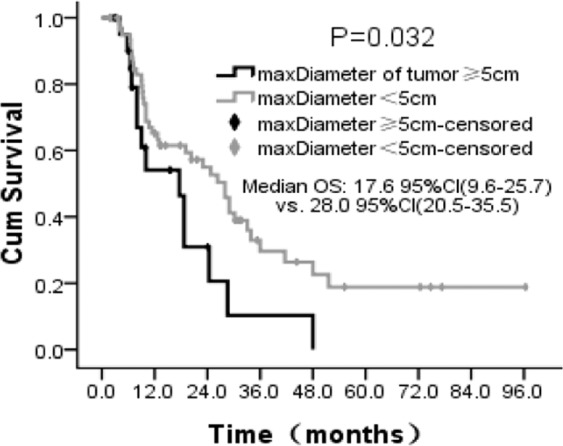
Overall survival probability of patients. Overall survival depending on the maximum diameter of AGM. Cum, cumulative.

Superior OS was only found in patients with the maximum diameter of AGM < 5 cm than those of ≥5 cm (28.0 months [95% CI: 20.5–35.5 months] vs. 17.6 months [95% CI: 9.6–25.7 months], P = 0.032) (Fig. 4). No significant correlation was found between OS and BED ≥ 80 Gy (P = 0.541), prescription dose≥45 Gy (P = 0.260), maxDose≥55 Gy (P = 0.367), number of AGMs (P = 0.786), lung primaries (P = 0.767), with simultaneous SBRT of other sites (P = 0.748), without other site of metastasis (P = 0.632), with systemic therapy (P = 0.559), or tracking method (P = 0.780).

For patients with tumor-induced symptoms before SBRT (including abdominal pain (n = 20), low back pain (n = 16), all symptoms (100.0%) were alleviated after SBRT.

### Adverse effects

Patients were all tolerant of SBRT. There were no grade 4 or more toxicities. Fatigue, nausea, poor appetite, pain, vommiting and leucopenia were the most common toxicities. Only 1 patient appeared with grade 3 diarrhea and 1 patient appeared with grade 3 fatigue. All patients recovered from the adverse effects after symptomatic treatment.

## Discussion

The study investigated the employment of SBRT in the management of AGM, which elucidated potential survival benefits and good LC with acceptable toxicities. Notably, an excellent symptom relief was found after SBRT, which may be attributable to its precise dosimetric distribution. Therefore, it is indicated that SBRT is suitable for treatment of AGM, especially for alleviations of various metastases-induced symptoms.

Recent studies have reported clinical practices of SBRT for different metastases, including the lung, liver, iliac lymph node, brain and bone metastases^15–17^. Nevertheless, the efficacy of SBRT for AGM has been evaluated in only a few studies^18–31^. The relevant studies in the past three years have been listed in Table 3. In our previous study^18^, 30 lung cancer patients with 32 AGMs received SBRT. Compared with the previous results, the LC rates were higher than that of this study, but with similar OS rates and slightly poorer PFS rates. Although the 30 lung cancer patients with AGMs in this study were the same as the previous published study, this study added more value. First, this study included more patients (75). Second, the origins of primary tumors were different. The previous published study only focused on SBRT for AGMs from lung cancer, while various origins of primary tumors in this study, including lung, liver, kidney, colorectal, stomach, esophageal, breast, nasopharyngeal, pancreatic cancer, non-Hodgkin lymphoma and adrenal cortical adenocarcinoma. Third, the last time of the follow-up was different. More importantly, more clinical characteristics were included in the analysis for investigations on potential correlations of OS and LC in this study. For example, we analyzed more prognostic factor about LC rate, maxDose, number of AGMs, different primary, other site metastasis, other site simultaneous SBRT and tumor size. And we further analyzed more prognostic factor about OS rate than that study, for example, different primary, tumor size, prescription dose, number of AGMs and so on. Thus, we could better screen indications from different angles for SBRT in the treatment of AGMs, not only the primary.

**Table 3:** Previous studies on SBRT for AGM in the past three years.

Study (year)	Patients (lesions)	Primary tumor	Dose	Overall survival	Local control	Toxicity
Zhao *et al*.^18^	30 (32)	Lung cancer	32–50 Gy/ 3–8 f	1-year: 58.1%2-year: 54.0%	1-year: 96.9%2-year: 72.7%	only 1 patient reporting grade-3 diarrhea
Katoh *et al*.^22^ 2018	20 (21)	45% NSCLC, 25% liver, 20% kidney, 5% bladder, 5% prostate	48 Gy/8 f (13 lesions), 40–50 Gy/5–8 f or 60–70 Gy/10 f (8 lesions)	1-year: 78.5%2-year: 45.8%	1-year: 91.7%2-year: 53.0%	No grade 3 or more acute and late toxicity
Burjakow *et al*.^23^ 2018	33 (38)	51.5% NSCLC, 6.1% SCC, 24.2% melanoma, 3% breast, 6.1% esophagus, 6.1% colorectum	48 Gy (28–68 Gy)/9–27 f	Median 11 mo	Median 21 mo1-year: 56.3%2-year: 50%	No grade 3 or above acute or late toxicity
Toesca *et al*.^24^ 2018	35 (39)	48% NSCLC, 20% liver, 9% gastrointestinal tract, 6% kidney, 17% other	40 Gy (20–54 Gy)/1–6 f	Median 19 mo	1-year: 92.4%2-year: 80.8%	No grade 3 or above acute or late toxicity
Plichta *et al*.^26^ 2017	10 (10)	50% NSCLC, 10% SCLC, 10% liver, 10% esophagus, 10% leiomyosarcoma, 10% unknown	30–48 Gy/3–5 f	Median 9.9 mo	1-year: 90%	No grade 3 or above acute or late toxicity
Haidenberger *et al*.^28^ 2017	23 (24)	39.1% NSCLC, 8.7% SCLC, 30.4% kidney, 4.3% liver, 4.3% breast, 4.3% pancreas, 4.3% melanoma, 4.3% unknown	19 patients: 20–25 Gy/f4 patients: 36–45 Gy/3 f	Median 29.2 mo1-year: 77%2-year: 72%	1-year: 95%2-year: 81%	No grade 3 or above acute or late toxicity
Chance *et al*.^29^ 2017	43 (49)	69.9% NSCLC, 14% SCLC, 4.6% ovary, 4.6% esophagus, 2.3% bladder, 2.3% breast, 2.3% skin	60 Gy (40–70 Gy)/4–15 f	Median 19 mo1-year: 65%2-year: 42%	1-year: 74%2-year: 57%	No grade 3 or above acute or late toxicity
Franzese *et al*.^30^ 2017	46 (46)	65.2% lung, 15.2% colorectum, 19.6% other	40 Gy/4 f	Mean 28.5 ± 1.6 mo1-year: 87.6 ± 6.1%2-year: 87.6 ± 6.1%	Median 14.5 ± 2.0 mo1-year: 65.5 ± 11.9%2-year: 40.7 ± 15.8%	No grade 3 or above acute or late toxicity

In another relevant report from The University of Rochester^19^, SBRT was performed in 30 patients with AGMs mostly from lung cancer, with dose schedules of 16~50 Gy in 4~10 fractions. The 1-year LC, OS, and distant control (DC) rate was 55%, 44%, and 13%, respectively. No≥2 grade adverse reaction was observed. Ippolito *et al*.^20^ reviewed 7 studies including 122 patients with AGM. Overall, the results of different studies varied widely (1-year LC rate: 44%~100%, 2-year LC rate: 27%~100%, 1-year OS rate: 58.1%~87.6%, 2-year OS rate: 42%~87.6%). It was reported a 2-year LC rate of more than 70% in about 50% of all studies. Distant metastasis was an important factor which affected the prognosis. The results in our study were better than those of most previous studies. Moreover, it was the first multicenter study to evaluate the efficacy and safety of SBRT as a main treatment option for AGMs. In addition to SBRT for AGM, other studies had also been summarized in Table 3.

Possibly, recent randomized trials presented in 2018 ASTRO conference elucidated potential survival benefits from SBRT for oligometastases, including AGMs, over palliative care alone. Palma *et al*. enrolled patients with 1–5 metastatic lesions, who were randomly allocated into palliative standard of care treatment group or palliative standard of care treatment plus SBRT to all metastatic lesions. It was demonstrated that both of OS and PFS were superior in palliative care plus SBRT than those of palliative treatment alone. There was no difference of QOL at 6 months during treatment between the two arms^32^. Another prospective trial from MDACC showed that compared to maintenance/observation therapy, local consolidative therapy improved OS and PFS in oligometastatic NSCLC^33^. Therefore, in addition to the previous retrospective studies, prospective and randomized trials also identified survival benefits from SBRT for metastatic lesions, including adrenal metastases.

Systemic therapies should be given as first priority in case of disease progression, especially tumor metastases. However, systemic therapy alone may not be adequate for all lesions, thus limited survival benefits were observed. Local consolidative therapy, including radiotherapy or other ablative therapy, combined with systemic therapy could provide synergistic effects. This result was similar to previous studies about liver or iliac lymph node metastases^16,17,21^. Wang *et al*.^17^ analyzed the clinical efficacy of SBRT for iliac lymph node metastasis. The results have found a longer OS in patients with systemic therapy. However, our study showed that patients with systemic treatment before or/and after treatment didn’t have a better OS but a better LC than those who had not. It may be ascribed to that patients with systemic treatment had multiple metastases while those without systemic treatment had solitary or limited metastases. Albeit the different chemotherapy regimens and time periods of chemotherapy between our study and previous studies, potential benefits from chemotherapy can’t be ignored, which requires further evaluations.

Moreover, published studies indicate that a BED_10_ ≥ 100 Gy was necessary to control primary NSCLC^34,35^. In our study, better LC was found in patients with BED_10_ more than 80 Gy with prescription dose more than 45 Gy. Therefore, a dose of 45 Gy should be prescribed in the case of 5 fractionations. Meanwhile, protection of normal organs was pivotal during prescription of doses.

Different origins of primary tumors might influence the efficacy of SBRT. Takeda *et al*. showed that the origins were independent factors of survival^36^. It was clarified that worse LC rates were found in metastases from colorectal cancer than those of metastatic lesions from other cancers. However, our previous study demonstrated that patients with iliac lymph node metastases from different origins had similar OS and LC^17^. In this study, no correlation was found in LC and OS between different origins of primary tumor, and the most common origins in our study were the lung (40.0%) and liver (26.7%). Despite of more rapid ameliorations of symptoms in patients with AGM from lung cancer, their prognosis was similar to those with AGM from other cancer (P = 0.169). The controversy may be attributable to the difference in tumor differentiation and radiosensitivity of AGM. Hence, it might be indicated that tumor origin may not be associated with the efficacy of SBRT and prognosis, rendering SBRT as an appropriate modality for metastases regardless of their origins. Nevertheless, the result should be confirmed in further studies.

In our study, the most two commonest symptoms resulting from AGM were abdominal pain and lumbar back pain. All patients had alleviations of symptoms after SBRT. Clawla *et al*.^19^ reported that all 3 patients with AGM were free from pain on a 10-point scale compared to 4–5 before radiotherapy, which was consistent with our research. As a result, it may be implied that earlier clinical practice of SBRT, if deemed possible at the initial diagnosis of AGM, may prevent the potential symptoms due to its excellent symptom relief effect.

Some limitations in our study were inevitable. Due to the retrospective manner, heterogeneity of treatment regimens was inevitable. Additionally, longer follow-up is necessary to further analyze the prognosis of SBRT and whether good LC could translate into survival benefits.

## Conclusion

SBRT is a promising modality for AGM for its good LC and amelioration of symptoms and mild radiation-induced toxicities, irrespective of the origin of primary tumor. Notably, it is required that systemic therapy, in addition to SBRT, should be also performed for an improved survival regarding distant metastases. Moreover, a radiation dose of more than 45 Gy or BED_10_ more than 80 Gy should be prescribed for better LC, without compromising protections of organs at risk. Prospective studies are required to further evaluate the correlation between doses and survival and the outcomes after SBRT.

## Supplementary information

Supplementary Table 1.
