# Supplementary material for: Clinical efficacy of Stereotactic Body Radiation Therapy (SBRT) for adrenal gland metastases: A multi-center retrospective study from China
Source: Sci Rep. 2020 May 12;10:7836. doi: 10.1038/s41598-020-64770-2 (PMC7217854; doi:10.1038/s41598-020-64770-2)
Supplement: Supplementary file 1 — Supplementary Table 1. [file 41598_2020_64770_MOESM1_ESM.doc]

# **Title**

**Clinical efficacy of Stereotactic Body Radiation Therapy (SBRT) for adrenal gland metastases: A multi-center retrospective study from China**

# **Author’s:**

Xianzhi ZHAO^1*^, Xiaofei ZHU^1*^, Hongqing ZHUANG^2^, Xueling GUO^1^, Yongchun SONG^3^, Xiaoping JU^1^, Ping WANG^3^, Zhiyong YUAN^3#^, Huojun ZHANG^1#^

1. Department of Radiation Oncology, Shanghai Changhai Hospital, Naval Medical University, Shanghai 200433, China.
2. Department of Radiotherapy, Peking University Third Hospital, Beijing 100191, China.
3. Department of Radiation Oncology and CyberKnife Center, Key Laboratory of Cancer Prevention and Therapy, Tianjin’s Clinical Research Center for Cancer, Tianjin Medical University Cancer Institute and Hospital, National Clinical Research Center for Cancer, Tianjin 300060, China.

* These authors contributed equally to this work.

^#^ Corresponding authors:

Zhiyong YUAN (Email: zhiyong0524@163.com), and

Huojun ZHANG (Email: [chyyzhj@163.com](mailto:chyyzhj@163.com))

**Supplementary Table 1: The dose-volume constraints for organs at risk (based on 5-fractionation regimen)**

| Parameter | Constraint/target |
| --- | --- |
| Renal hilum/vascular trunk | V23< 2/3 volume |
| Renal cortex (right and left) | V17.5< 200 mL |
| Duodenum | V5mL < 18 Gy, V10mL < 12.5 Gy and Dmax< 32 Gy |
| Liver | V21< 700 mL |
| Spinal cord | V0.35mL < 23 Gy, V1.2mL < 14.5 Gy and Dmax< 30 Gy |
| Medulla spinal cord(5-6mm above and below level treated per Ryu) | V23< 10% of subvolume and Dmax< 30 Gy |
| Stomach | V10mL < 18 Gy and Dmax< 32 Gy |
